# Neurostimulation Treatment in Chronic Cluster Headache—a Narrative Review

**DOI:** 10.1007/s11916-021-00989-6

**Published:** 2021-12-11

**Authors:** Stefan Evers, Oliver Summ

**Affiliations:** 1 Faculty of Medicine, University of Münster, Münster, Germany; 2 Lindenbrunn Hospital, Coppenbrügge, Germany; 3 Department of Neurology and Research Center of Neurosensory Science, Carl Von Ossietzky University, Oldenburg, Germany; 4 Department of Neurological Intensive Care and Rehabilitation, Evangelisches Krankenhaus Oldenburg, Oldenburg, Germany

**Keywords:** Cluster headache, Deep brain stimulation, Vagus nerve stimulation, Sphenopalatine ganglion stimulation, Greater occipital nerve stimulation

## Abstract

**Purpose of Review:**

In this narrative review, the current literature on neurostimulation methods in the treatment of chronic cluster headache is evaluated. These neurostimulation methods include deep brain stimulation, vagus nerve stimulation, greater occipital nerve stimulation, sphenopalatine ganglion stimulation, transcranial magnetic stimulation, transcranial direct current stimulation, supraorbital nerve stimulation, and cervical spinal cord stimulation.

**Recent Findings:**

Altogether, only nVNS and SPG stimulation are supported by at least one positive sham-controlled clinical trial for preventive and acute attack (only SPG stimulation) treatment. Other clinical trials either did not control at all or controlled by differences in the stimulation technique itself but not by a sham-control. Case series report higher responder rates.

**Summary:**

The evidence for these neurostimulation methods in the treatment of chronic cluster headache is poor and in part contradictive. However, except deep brain stimulation, tolerability and safety of these methods are good so that in refractory situations application might be justified in individual cases.

## Introduction

Neurostimulation has become a major focus in the treatment research on idiopathic headache disorders. In particular, migraine and cluster headache have been studied whether these disorders respond to different types of neurostimulation (with respect to both stimulation technique and anatomical region). In this narrative review, we aim to give a brief clinical summary of the published evidence for different stimulation types in chronic cluster headache (CCH). The approach of neurostimulation in cluster headache is however not really new. Already in 1950, an experiment was published showing that stimulation of nerve fibres of the sphenopalatine ganglion in provoked cluster headache attacks was efficacious [1].

When reviewing neurostimulation in CCH, the following aspects have to be considered:

- Stimulation technique
- Acute or preventive treatment
- Site of stimulation

We decided to structure our review according to the site of stimulation since this is in concordance with the major clinical trials.

## Deep Brain Stimulation

Deep brain stimulation (DBS) of the ipsilateral posterior hypothalamus was the first specific stimulation technique published on the treatment of refractory CCH [2]. This technique was developed following the neuroimaging finding that activation of the posterior hypothalamus plays a major role in cluster headache attacks [3]. Since the first published positive case report [2], several case series with number of patients between 4 and 21 have been published until now, reporting an efficacy in CCH prevention with more than 50% responders [4•, 5–13]. All reports differ slightly with respect to the exact anatomical localisation (major alternative sites are the ventral tegmental area; midbrain ventral and retrorubral tegmentum; endoventricular site of the third ventricle wall; dorsal longitudinal and mamillotegmental fasciculi) and with respect to the exact parameters of the stimulation.

The effects of DBS seem to remain stable in most patients over a long time. One observational study followed patients with DBS (*n* = 7) and patients with greater occipital nerve (GON) stimulation (*n* = 17) over 48 months [13]. All the patients from the DBS group were considered responders at final follow-up, with more than 85% being satisfied with the treatment; approximately 29% of initial responders to GON stimulation became resistant at the final follow-up. A meta-analysis of published cases (*n* = 40) found a significant 77% mean reduction in headache attack frequency over a mean follow-up of 44 months, with an overall response rate of 75% [14]; another case series with an observation period over 8.7 years showed also consistent efficacy of DBS in CCH in 70% without side effects (15Leone 2013). Positive outcome was not associated with demographic covariates, negative outcome was associated with bilateral cluster headache. The findings confirming long-term effectiveness of DBS for CCH suggest that the neuroanatomical substrate of deep brain stimulation-induced headache relief is probably not restricted to the posterior hypothalamic area but encompasses a more widespread area in the midbrain.

Acute CCH attack treatment by DBS was also studied, but only 23% of all attacks in a larger case series were improved by direct DBS stimulation; therefore, DBS cannot be regarded as effective in the treatment of attacks [16].

The enthusiasm for this technique decreased when an intracerebral haemorrhage due to the implantation procedure was reported [5]. Another setback for the development of this technique was the first randomised, prospective, crossover, double-blind study assessing the efficacy and safety of ipsilateral hypothalamic DBS in 11 patients with severe refractory CCH [17•]. During the randomised phase, no significant change in primary and secondary outcome measures was observed between active and sham stimulation. There were three serious adverse events, including subcutaneous infection, transient loss of consciousness and micturition syncopes. It has also been shown that DBS in CCH does not result in a change of cognition [18].

DBS has also been applied in other trigemino-autonomic cephalalgias such as short-lasting unilateral neuralgiform headache attacks with conjunctival injection and tearing (SUNCT; *n* = 2) and for chronic paroxysmal hemicranias (*n* = 1) [19]. In these cases, the contralateral hypothalamus was (also) stimulated.

## Vagus Nerve Stimulation

Vagus nerve stimulation has been studied as non-invasive (transdermal) stimulation at the neck (along the carotid artery) for migraine and cluster headache; the common abbreviation for this technique is nVNS. It can be used as acute attack treatment or as preventive treatment by regular daily stimulation. A small observational study with 18 patients including 11 with CCH showed an improvement by 50% for both acute and preventive treatment [20].

In a prospective, open-label, randomised study in CCH patients, add-on prophylactic nVNS (*n* = 48) was compared with standard of care (SoC) alone (*n* = 49) [21•]. During the randomised phase, individuals in the intent-to-treat population treated with SoC plus nVNS had a significantly greater reduction in the number of attacks per week than control patients (−5.9 versus −2.1). The rate of responders (> 50% reduction) was also higher in SoC plus nVNS (40%) than in control patients (8.3%). No serious treatment-related adverse events occurred.

A retrospective observational study in the UK showed a mean CCH attack frequency from 26.6 + / − 17.1 attacks per week before initiation of nVNS therapy to 9.5 + / − 11.0 attacks per week (*p* < 0.01) afterwards. Also, attack duration, attack severity, and use of abortive treatments decreased [22].

The efficacy of nVNS on acute attacks in cluster headache has been studied in two randomised sham-controlled studies [23, 24•]. Interestingly, both studies showed that acute attack treatment by nVNS has significant efficacy in attack abortion in episodic cluster headache attacks but not in CCH attacks. Interestingly, however, it has also been shown that nVNS is cost-effective in CCH, at least for acute attack treatment [25].

## Greater Occipital Nerve Stimulation

Greater occipital nerve (GON) stimulation with implanted electrodes in CCH has first been reported in 2007 with a case series of 8 patients responding between 25 and 95% attack frequency reduction [26]; the same group confirmed this result in a larger case series of 18 patients with medically intractable cluster headache [27]. Other case series reported a substantial effect of GON stimulation in 7 out of 8 patients [28] and in 9 out of 10 patients [29]. A mean attack frequency and intensity decrease by 68% and 49%, respectively, was observed in 13 patients [30], local infection occurred in one patient, leading to hardware removal. Another large case series on GON stimulation in CCH (51 patients) reported at least 50% improvement of attack frequency in 53% of patients [31]. In a long-term observational study with a median follow-up of 6.1 years, 67% of the patients were responders (≥ 50% reduction in headache attacks per day) and 40% responders showed a stable condition characterised by only sporadic attacks [32].

A recent and large (*n* = 105) observational study showed an attack frequency reduction by > 50% in 69% of the patients [33•]. Mean weekly attack frequency decreased from 22.5 at baseline to 9.9 after GON stimulation. Preventive and abortive medications were significantly decreased. When comparing baseline and 1-year and last follow-up outcomes, efficacy was sustained over time. During the follow-up, 67 patients experienced at least one complication, 29 requiring an additional surgery: infection (6%), lead migration (12%) or fracture (4.5%), hardware dysfunction (8.2%), and local pain (20%).

Most recently, a randomised, double-blind, electrical dose-controlled clinical trial has been performed showing an efficacy of both 100% and 30% GON stimulation intensity in 130 patients with CCH with no significant difference between the two stimulation intensities [34•]. A median decrease of CCH attacks by 4.1 per week in the 100% stimulation group and by 6.5 per week for the 30% stimulation group was observed. The most common adverse events were local pain, impaired wound healing, neck stiffness, and hardware damage. This study was interpreted as positive although there was no sham control.

GON stimulation for the treatment of refractory CCH and chronic migraine has been regarded as a cost-intensive treatment option with a significant complication rate [35]. However, occipital nerve stimulation for refractory CCH is the only available invasive approach with a Conformité Européenne (CE) mark.

An alternative technique to GON invasive stimulation is subcutaneous GON stimulation which has however only been reported in small case series by the same group [36, 37]. Another alternative technique is bilateral burst stimulation of the GON which produces less paresthesia. The efficacy of this technique in CCH has only been shown in a small case series of *n* = 5 [38].

## Sphenopalatine Ganglion Stimulation

A further target for electrical stimulation is the sphenopalatine ganglion (SPG). Clinically, the involvement of the SPG in the pathophysiology of trigeminoautonomic headache is evident by symptoms as lacrimation, nasal congestion or rhinorrhea. These symptoms are driven by nerval activation of parasympathic fibres that synapse in the SPG. Anatomically the SPG is located in the pterygopalatine fossa and comprises the parasympathic synapse and parasympathetic cell bodies as well as sensory axons and sympathetic fibres which are post-synaptic.

Research in animal studies as well as in human studies gave further insights to the pathophysiological circuits of activity in regions as the hypothalamus, brainstem and activation of efferent parasympathic fibres with effect on afferent trigeminal fibres as it can be observed in the trigeminal autonomic reflex arc [3, 39–41]. Interestingly, pharmacological treatments with an effect on trigeminoautonomic cephalalgias were shown to substantially modulate autonomic outflow [41] in an animal model of trigeminal cephalalgias, underlining SPG as a target for treatment of trigeminoautonomic headaches. Applied in humans, low frequency stimulation of the SPG is able to provoke autonomic symptoms attributed to an activation of the parasympathic outflow from the SPG through the applied low frequency stimulation, whilst high frequency stimulation is attributed to a blockage of the parasympathic outflow [42, 43]. Contrary to the initial trial [42] cluster like headaches were not demonstrated being triggered by low-frequency stimulation of the SPG in a double-blind randomised sham-controlled crossover study [43].

Favourable results of SPG stimulation, applied as pulsed treatment either during attacks or repeatedly on daily basis, were reported in an open label follow-up study of the Pathway CH-1 study in which 33 patients with CCH were followed up for 24 months [44]. In this study, it has been demonstrated that in 30% of these patients a minimum of one period of attack remission was observed beginning after a treatment period of about 3.5 months, also the HIT-6 score as a marker for the headache disability improved by an average of 12.5 points. In another report of this group, 45% of the patients were described as being acute responders at 24 months and 33% patients responded in terms of a frequency reduction, drawn together the total response observed was 61% [45]. Summarised in the most recent report of this group, 88 CCH patients with SPG stimulation of which 78 stayed implanted were included and followed up for 12 months, all these patients were responders by measures of HIT-6 and 67% responded in the SF-36 [46]. A frequency reduction was seen in 55%, 32% were acute responders, and an overall response was documented in 68%. Seventy-four percent of the CCH patients were even able to stop, reduce, or remain of all preventive medication. In a smaller case series, 11 out of 18 attacks in patients with intractable CCH were completely aborted by SPG stimulation [47].

The observation of 36 patients in a SPG stimulated group versus 40 sham-stimulated patients in a US-based randomised, sham-controlled, parallel group, double-blind, safety and efficacy study, demonstrated an even higher rate of acute responders [48••]. Pain relief, defined as reduction from a minimum of moderate pain to none or mild pain, experienced at 15 min poststimulation was documented in 62%, whilst the response in terms of median frequency reduction was at least 75%. Adverse events most commonly seen after implantation of SPG stimulator and concomitant stimulation were sensory disturbances (with stimulation), headache, and pain [48••, 49].

The approach of pulsed radiofrequency of the SPG in patients with CCH is another invasive option for the treatment. Case reports and series report on different outcomes, e.g. it led to unsuccessful treatment of the pain as well as the autonomic function in three reported patients, whereas they were reported being successfully treated by thermocoagulation of the SPG, though the follow-up ended after 11 month [50]. However, in a prospective analysis of the treatment with radiofrequency ablation or pulsed radiofrequency, 37 patients with CCH were followed up by a mean of 68 months, only 30% of the patients were reported not having improved but five of the patients even demonstrated a total relief of headache and autonomic symptoms [51]. It will have to be demonstrated in further comparisons which technique will be more successful in the treatment of pain and safer for the patient as there has been shown a low complication rate reported [51] in comparison to stimulation of the SPG.

## Transcranial Magnetic Stimulation

Transcranial magnetic stimulation (TMS) offers an interesting opportunity as it is a well-established technique for other indications that can be applied safely without direct stimulation via implants and therefore potential adverse events as infection or migration of implant as well as the necessity of exchange of an energy source are avoided. TMS is able to generate a small electric current on the cortex area stimulated through electromagnetic induction that is highly specific to a defined area in dependence to the parameters, i.e. single-pulse TMS, high- or low-frequency repetitive TMS, and configuration of the stimulation coil used. TMS was demonstrated being able to inhibit the propagation of cortical spreading depression (CSD) which is accepted as the pathophysiological correlate of migraine aura thus its role in migraine without aura still is under debate [52].

With a focus on treatment of headache symptoms, positive studies on the application of TMS are evident for the blockade of CSD in animal studies [52]. Single-pulse TMS applied at the occiput has demonstrated effectiveness in acute treatment of migraine pain in patients suffering from migraine with aura [53], paralleled by a migraine preventive effect [54] that is also observed in repetitive TMS over the M1 area [55]. Taken together, the data from the available studies led to the rating as moderate recommendable for migraine pain prevention, and recommendable for migraine prevention in terms of reduction of headache days, by the International and the North American Neuromodulation Society (INS and NANDS) [56].

In CCH, however, no recommendation can be made for TMS treatment, as there is a lack of randomised controlled trials, though an open-label observational study including 19 patients suffering from CCH reported a beneficial outcome of repetitive (10 Hz) TMS over the M1 area. Paroxysmal pain and number of attacks were reduced when comparing baseline with 15 days poststimulation measures [57]. Although in this report permanent pain was reported in 8 of the cluster headache patients, it remains unclear if those fulfilled the definition of CCH; therefore, no clear data are available on the use of TMS in CCH.

## Transcranial Direct Current Stimulation

Transcranial direct current stimulation (tDCS) (anode at Fz, cathode over C7) for refractory CCH patients has only been studied in a proof-of-concept study [58] with a mean attack frequency reduction by 35%. The method was well tolerated. There are no more data available on this method in CCH at the moment.

## Supraorbital Nerve Stimulation

The stimulation of the supraorbital nerve in CCH patients has only been described in case reports. These described positive responses in the patients [59, 60]; no more data on this technique as a single treatment is available.

## Cervical Spinal Cord Stimulation

One case series described the efficacy of high spinal cord stimulation in refractory CCH patients [61]. All seven patients showed significant treatment effects with immediate improvement after electrode implantation. This was confirmed by a single case report of CCH [62].

## Conclusion

This narrative review shows that there is a variety of techniques which has been applied to patients with CCH. Most of these techniques were detected empirically; however, DBS and SPG stimulation follow our current knowledge on the pathophysiology of cluster headache. In summary, nearly all techniques are safe and well tolerated. Only DBS electrodes should be implanted with very high caution since fatal outcome of the operation has been reported. The highest rate of technical problems after implantation has been reported for GON stimulation.

The efficacy of all stimulation procedures has been reported as quite high, at least in the open case series and observational studies. The randomised trials, if any, showed poorer results. We summarised the results for the different stimulation techniques and the preventive and acute treatment in a subjective way (Table 1). However, this overview shows that only nVNS and SPG stimulation are supported by at least one positive sham-controlled clinical trial. Other clinical trials either did not control at all or controlled by differences in the stimulation technique itself but not by a sham-control.

**Table 1:** Stimulation techniques and their efficacy in chronic cluster headache according to published evidence

Techniques	RCT	Open trials
Preventive treatment		
DBS	One trial negative	Several case series positive; consistent efficacy over years
nVNS	One trial positive	Some case series positive
GON stimulation	One trial positive without sham	Several case series positive
SPG stimulation	One trial positive	Larger case series positive
SPG radiofrequency	None	Two small case series positive
TMS	None	One small case series positive
Acute treatment		
DBS	None	One small case series negative
nVNS	Two trials negative	Only reports
SPG stimulation	Two trials positive	Small case series positive

The real efficacy of the stimulation techniques remains also unknown since only refractory patients were treated (and included in trials). It might be that patients responding to oral drugs also respond to stimulation techniques. It remains to be debated whether neurostimulation should be restricted only to refractory CCH patients.

One option for future applications might be the combination of different stimulation techniques, as it has been tried in migraine. Only one case report combining DBS and nVNS [63] and one case report combining GON stimulation with supraorbital and infraorbital nerve stimulation [64] are available. Another approach is the sequential application of the different techniques described above. This has been tested in an observational study on 44 patients with CCH who received first SPG stimulation, then (if SPG stimulation was ineffective) GON stimulation and then (if GON stimulation was ineffective) DBS stimulation resulting in a total responder rate of 93% [65].

In conclusion, there is a lot of open data but only very poor clinical evidence for the efficacy of neurostimulation techniques in the treatment of CCH. Since this condition is extremely disabling and often refractory to drugs, it is justified to try such stimulation techniques as last option. However, one should follow clinical evidence and safety concerns as reported in this review.
